# A Probabilistic Spatial Dengue Fever Risk Assessment by a Threshold-Based-Quantile Regression Method

**DOI:** 10.1371/journal.pone.0106334

**Published:** 2014-10-10

**Authors:** Chuan-Hung Chiu, Tzai-Hung Wen, Lung-Chang Chien, Hwa-Lung Yu

**Affiliations:** 1 Department of Bioenvironmental systems engineering, National Taiwan University, Taipei, Taiwan; 2 Department of Geography, National Taiwan University, Taipei, Taiwan; 3 Division of Biostatistics, University of Texas School of Public Health at San Antonio Regional Campus, San Antonio, Texas, United States of America; Research to Advance Community Health Center, University of Texas Health Science Center at San Antonio Regional Campus, San Antonio, Texas, United States of America; INDEPTH Network, Ghana

## Abstract

Understanding the spatial characteristics of dengue fever (DF) incidences is crucial for governmental agencies to implement effective disease control strategies. We investigated the associations between environmental and socioeconomic factors and DF geographic distribution, are proposed a probabilistic risk assessment approach that uses threshold-based quantile regression to identify the significant risk factors for DF transmission and estimate the spatial distribution of DF risk regarding full probability distributions. To interpret risk, return period was also included to characterize the frequency pattern of DF geographic occurrences. The study area included old Kaohsiung City and Fongshan District, two areas in Taiwan that have been affected by severe DF infections in recent decades. Results indicated that water-related facilities, including canals and ditches, and various types of residential area, as well as the interactions between them, were significant factors that elevated DF risk. By contrast, the increase of per capita income and its associated interactions with residential areas mitigated the DF risk in the study area. Nonlinear associations between these factors and DF risk were present in various quantiles, implying that water-related factors characterized the underlying spatial patterns of DF, and high-density residential areas indicated the potential for high DF incidence (e.g., clustered infections). The spatial distributions of DF risks were assessed in terms of three distinct map presentations: expected incidence rates, incidence rates in various return periods, and return periods at distinct incidence rates. These probability-based spatial risk maps exhibited distinct DF risks associated with environmental factors, expressed as various DF magnitudes and occurrence probabilities across Kaohsiung, and can serve as a reference for local governmental agencies.

## Introduction

Dengue fever (DF) is among the most severe vector-borne infectious diseases spread by mosquitoes in tropical and subtropical regions; 2.5 billion people in over 100 countries are at risk of contracting the dengue virus [1]–[5]. More than 50 million infections occur annually, particularly in Southeast Asian and western Pacific regions [5], [6]. Several species of mosquito (e.g., *Aedes aegypti* and *Aedes albopictus*) transmit five distinct dengue virus serotypes, including DENV-1, DENV-2, DENV-3, and DENV-4 [7], [8]. No commercially available vaccine exists to mitigate the disease spread, and therefore, determining the spatiotemporal distribution of DF incidence is among the top priorities for DF prevention and control [9]–[13]. Although numerous DF studies have focused on determining the DF etiology to facilitate space–time prediction, a risk assessment framework to account for DF risk factors and provide risk measures across space (e.g., the probability and magnitude of DF incidences) must be established to control and manage the disease.

Identifying the major surrogate factors for DF incidence is key to assessing DF risk. Ideal surrogate factors are risk factors that not only are closely associated with DF etiology, but also should be readily available [13], i.e., easier to be accessed or observed For example, climatic variables are a critical factor characterizing the temporal patterns of DF incidence [14]. Regarding the spatial characteristics of DF incidences, previous research has explored land-use indicators and socioeconomic status to approximate the local magnitudes of complex interactions between infected and susceptible human hosts and DF vectors [15]–[18]. High-risk DF areas are closely associated with the size and location of agricultural, forest, and residential areas, which are the preferred habitats of DF vectors. Populated areas close to vector-preferred areas exhibit increased DF occurrence risk [15], [19]–[21]. In addition, previous studies have indicated that high population densities, residential areas, and areas with low income family exhibit increased risk of DF transmission [22]–[25]. The statistical relationships among DF incidence, land use, and socioeconomic factors frequently change across the study area because of the distinct environmental and climatic conditions. Therefore, determining the associations between DF outbreaks and the environmental factors of each study area is essential. We used quantile regression to characterize the relationships between risk factors and DF incidence across the quantiles of the DF incidence probability distribution.

Probabilistic risk assessment is a systematic approach to evaluating the risk of an event in terms of probabilities and their associated magnitudes [26]–[28]. Although a probabilistic approach provides a means to characterize the uncertainties of the events (e.g., a DF epidemic), interpreting the probability associated with risks is difficult for the general population and decision makers [29]. By contrast, frequency analysis is used to estimate the return period a prespecified event (e.g., extreme rainfall, floods, tornados, earthquakes) [30]. The return period is the average number of years between occurrences of the annual event with magnitude larger than a specified level. The selected and analyzed annual events should be homogeneous and independent [31]. In other words, interpreting the probabilities of risks is possible by estimating their associated return periods, using frequency analysis.

This paper proposes a threshold-based quantile regression approach to investigate the functional relationships between the spatial distributions of environmental risk factors, including land use and socioeconomic factors, and DF incidence. The functional relationship was characterized using estimations at prespecified quantiles of DF incidence (i.e., 0.1, 0.2,…, 0.9) that comprise the spatially varying nonparametric probability distribution of DF risk associated with the spatial factors. The spatial distributions of DF risk were estimated and expressed as return periods for ease of interpretation.

## Materials

### Study area

**Figure 1:**
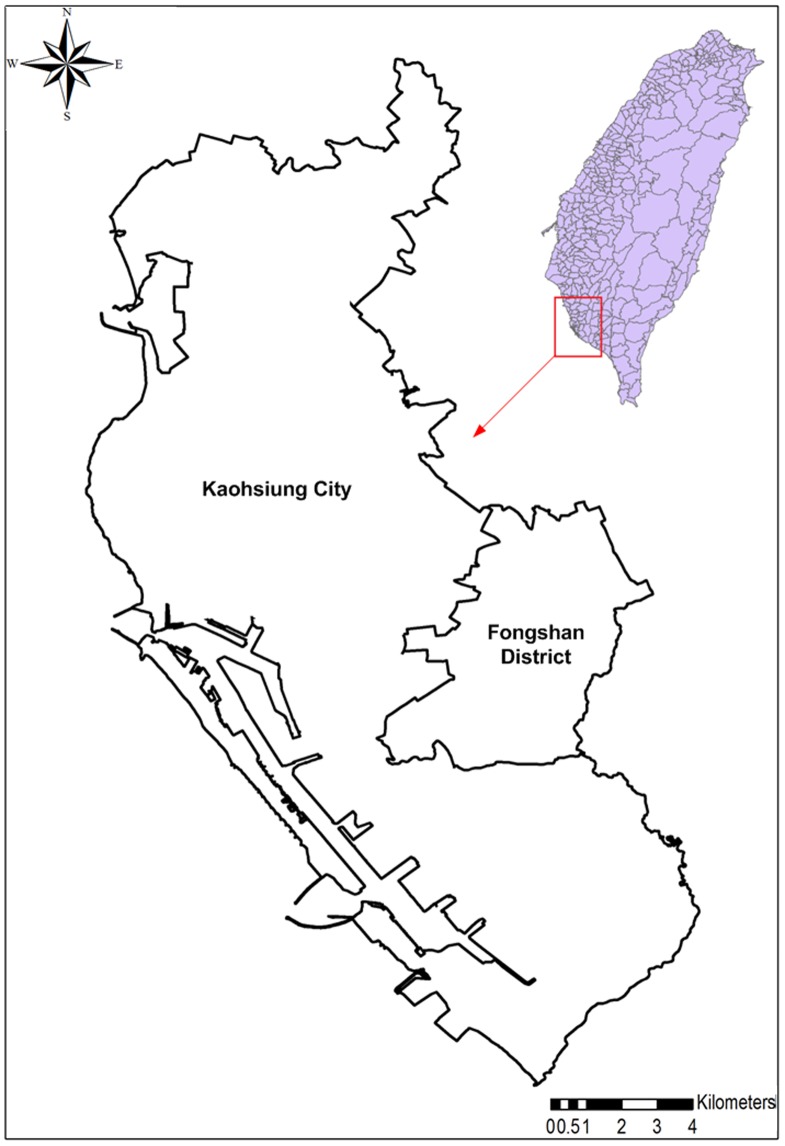
Map of Kaohsiung city and Fongshan district.

For decades, the DF annual incidence rate has been among the highest for infectious diseases in Taiwan [32] and is particularly high in old Kaohsiung City (Fig. 1), the second largest city in Taiwan with a population of 2.9 million distributed over 11 districts and an area of 154 km^2^. Fongshan District, located to the southeast of Kaohsiung City and covering an area of 26 km^2^, was the former capital of Kaohsiung County and has a population of approximately 36 000. Figure 1 depicts the area covered in this study: old Kaohsiung City and Fongshan District, which both include major commercial, residential, and political areas of Southern Taiwan. This area comprises 535 *li* (the smallest administrative unit in Taiwan) with population sizes ranging from 160 to 37 000 and an average population density of approximately 950 people per square kilometer (Fig. 1). The study area is located in a tropical region that has a clear distinction between dry and wet seasons. The wet season typically lasts for half a year, from mid-May to mid-October, during which time the daily rainfall can exceed 500 mm during typhoon periods. The dry season comprises the rest of the year, during which time there is little to no rain. The average annual rainfall of this area is approximately 1884.9 mm. The daily temperature exceeds 20°C for most of the year, with minimum and maximum average monthly temperatures of 19.3°C in January and 29.2°C in July, respectively. Because of the limited area (i.e., 180 km^2^) and topographical changes of the study area, the variability of climatic variables (e.g., temperature) across the study area was relatively small.

### Data

**Figure 2:**
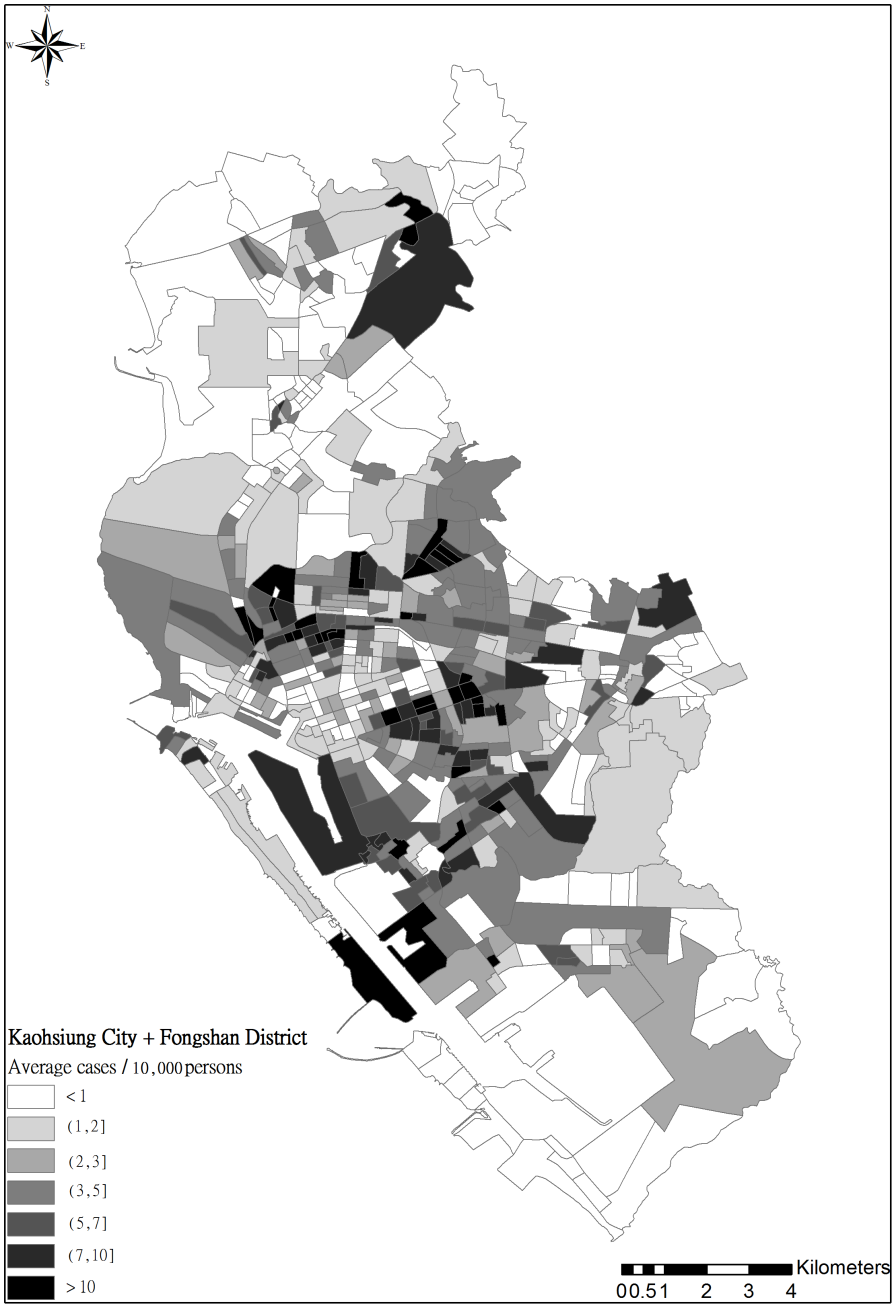
Spatial distribution of average DF incidence rate in 535 Li's during 2004–2011.

**Figure 3:**
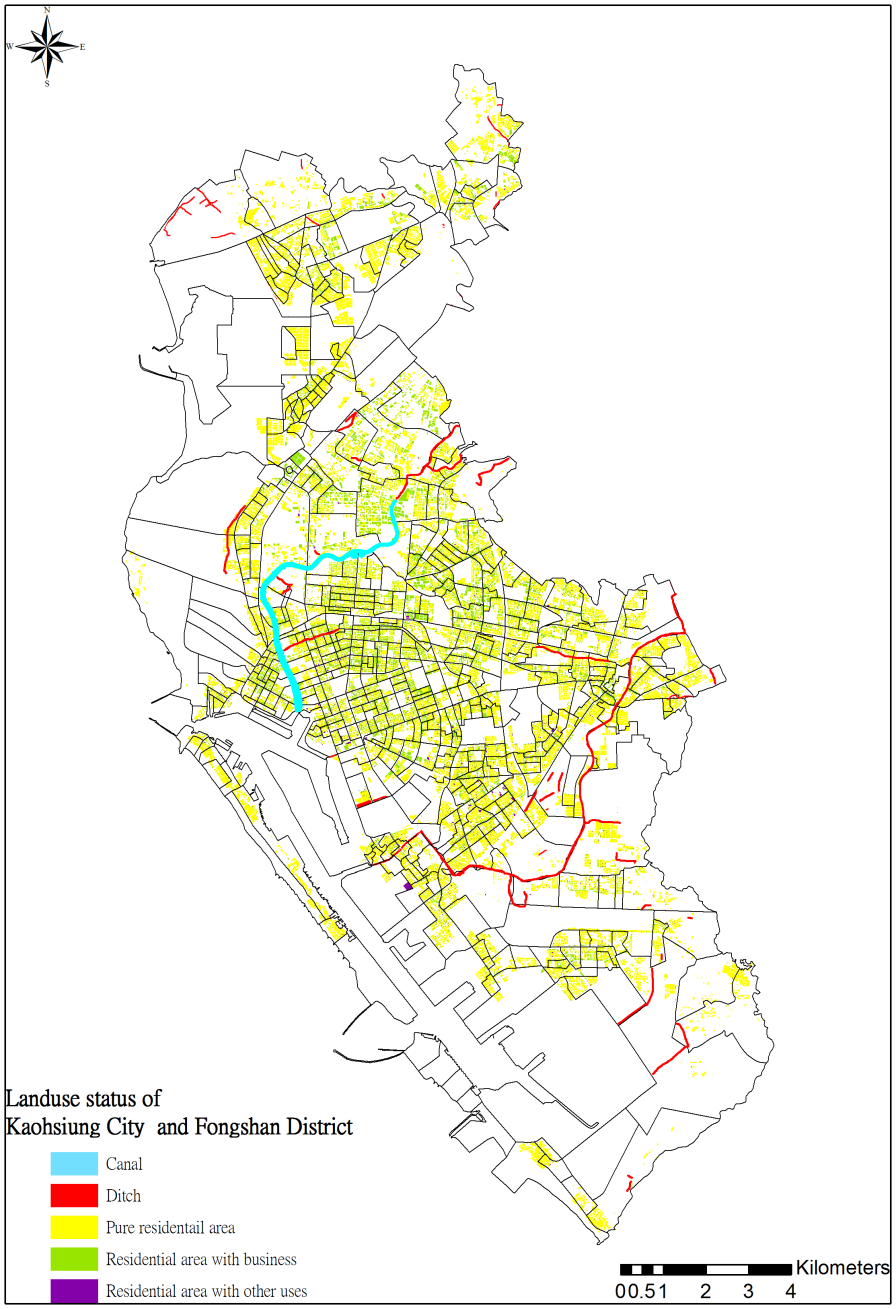
Spatial distribution of landuse factors important to DF across Kaohsiung-Fongshan area.

We investigated DF cases based on surveillance data obtained from the Centers for Disease Control (CDC) in Taiwan. Data was obtained by following the standard data request procedure on the CDC website [33]. The dataset included total DF cases in the 353-*li* area, including both local and imported cases, from 2004 to 2011. Only 2% of the total reported DF cases were considered imported in the dataset, and no significant distribution patterns of imported cases across space and time were observed. Figure 2 illustrates the spatial distribution of the average annual incidence of DF. The DF risk factors considered in this study included a various land uses and socioeconomic attributes. Land-use data were obtained from the Taiwan National Land Surveying and Mapping Center (NLSC) [34]. This analysis included 103 land-use factors, including agriculture, forest, public utility, and residential areas. The NLSC website lists a detailed description and definition of every land-use factor [35], [36]. Figure 3 shows various land-use factors (i.e., canals, ditches, and residential areas). The land-use factors for each *li* are expressed according to area ratio and size, i.e., the percentage and areal size of a land-use factor in the *li*'s. Socioeconomic data were collected from the Fiscal Information Agency of the Ministry of Finance in Taiwan in 2009. This dataset comprises the spatial distributions of 12 socioeconomic attributes, such as per capita income, and average population density across the 535 *li*.

## Method

Koenker and Bassett [37] first introduced quantile regression specifically to estimate the relationship between covariates and the quantile of response variable distribution. The regression is flexible, allowing covariates to exert various effects at distinct points of the distribution, and the estimation is robust to nonnormality and skewed tails of the data distribution [38]. We adopted quantile regression to investigate the underlying relationships between average annual DF incidence rate 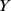 (i.e., average cases per 10 000 people) and potential risk factors 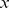 (i.e., land use and socioeconomic attributes). Unlike conventional regression approaches, quantile regression not only considers the central tendency of the response variable regarding covariate changes, but also determines the covariate effect conditional on specific quantiles of the response variable.

Let 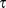 within the range of 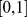 be a quantile level of the 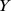 distribution. The relationship between 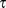 and 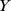 is denoted as 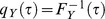, where 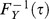 is an inverse of the cumulative distribution function (CDF) of 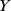. Given *li* location 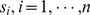, 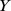 and 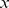 are rewritten as 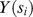 and 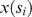, respectively. In addition, 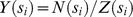, where 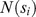 and 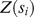 are average dengue cases and population size 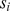, respectively. Thus, the relationship between 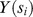 and 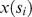 in selected quantile 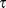 can be written as
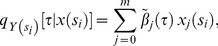
(1)where 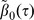,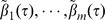 denotes the estimated intercept and coefficients in a prespecified quantile level 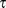, and 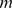 is the number of 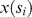. The DF dataset indicated the absence of DF incidence in some *li* during the study period, implying that the relationship between the covariate changes and dengue incidence quantiles were not linear; specifically, thresholds existed for the risk factors above which the factors significantly affected the DF risk change. Based on this assumption, the general quantile regression formula shown in Eq. (1) was transformed into a threshold-based quantile regression to account for the threshold values for each identified covariate. To obtain the model thresholds, the original dataset was classified into two sets, 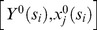 and 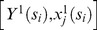, where 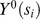 and 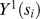 represent the observed average incident rate equal to zero and larger than zero, respectively, and 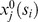 and 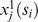 are their corresponding risk factors at location 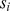. The proposed threshold-based quantile regression model can be expressed as



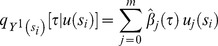
(2)where 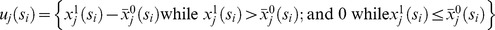; 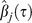, *j* = 1, …, *m* are the estimated coefficients for threshold-adjusted covariates 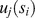; and 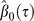 is the intercept. To determine the influential variables, a two-stage variable selection process (i.e., variable screening and stepwise variable selection) was employed. In the first stage, variables were removed as long as they are closely linear correlated with other variables and, based upon literature review, not evident to be a risk factor of DF incidence. In the variable screening process, before performing a quantile regression, a Pearson correlation, 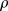, was implemented to evaluate collinearity among the risk factors, where 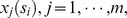 with absolute correlation coefficients larger than 0.35 with other covariates were removed before further analysis. For example, the variables of road areal ratio and population density were highly correlated (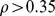). Road areal ratio was removed because population density exerted a greater influence on DF in studies [22], [24], [39]. In the variable selection process, the covariate selection in Eq. (2) followed the stepwise selection technique [40], based on Akaike's information criterion (AIC) [41] (i.e., the lower the AIC, the better the performance of the model is). The optimal thresholds 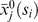 for each covariate were estimated by maximizing the pseudo *R^2^* (*pR^2^*). The *pR^2^* of the optimal models at various quantile levels were calculated separately to assess their performances. The analyses of the quantile regression models were performed using the quantreg package in R [42].

The semiparametric CDF for 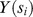 was estimated with respect to 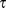
and 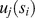 by using Eq. (2). The quantile-based CDF can be used to estimate the exceedance probability (EP) of 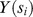, 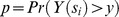, where 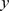 is a selected annual DF incidence rate. In frequency analysis, a distinct perspective of EP can be obtained by using return period due to the close relationship between the probability distribution and return period of an event. Assuming the yearly DF cases during the study period were independent, the return period of a specified annual incidence rate would be equal to the reciprocal of its EP (i.e., 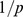) based on frequency analysis. For example, a 10-year DF outbreak has a 1/10 = 0.1 or 10% chance of being exceeded in one year. This approach facilitated a probabilistic risk assessment of DF epidemics by estimating the full CDFs of DF risk across the study area without distributional assumptions. DF risk spatial distributions can be expressed as DF incidence statistical characteristics (e.g., mean and median) or EP and return periods regarding the prespecified EP and incidence rates. Based on risk maps, areas of dense DF incidence (e.g., locations with high average incidence rates or short return periods) can be identified. This approach can encourage public health authorities to prioritize DF prevention.

## Results

**Figure 4:**
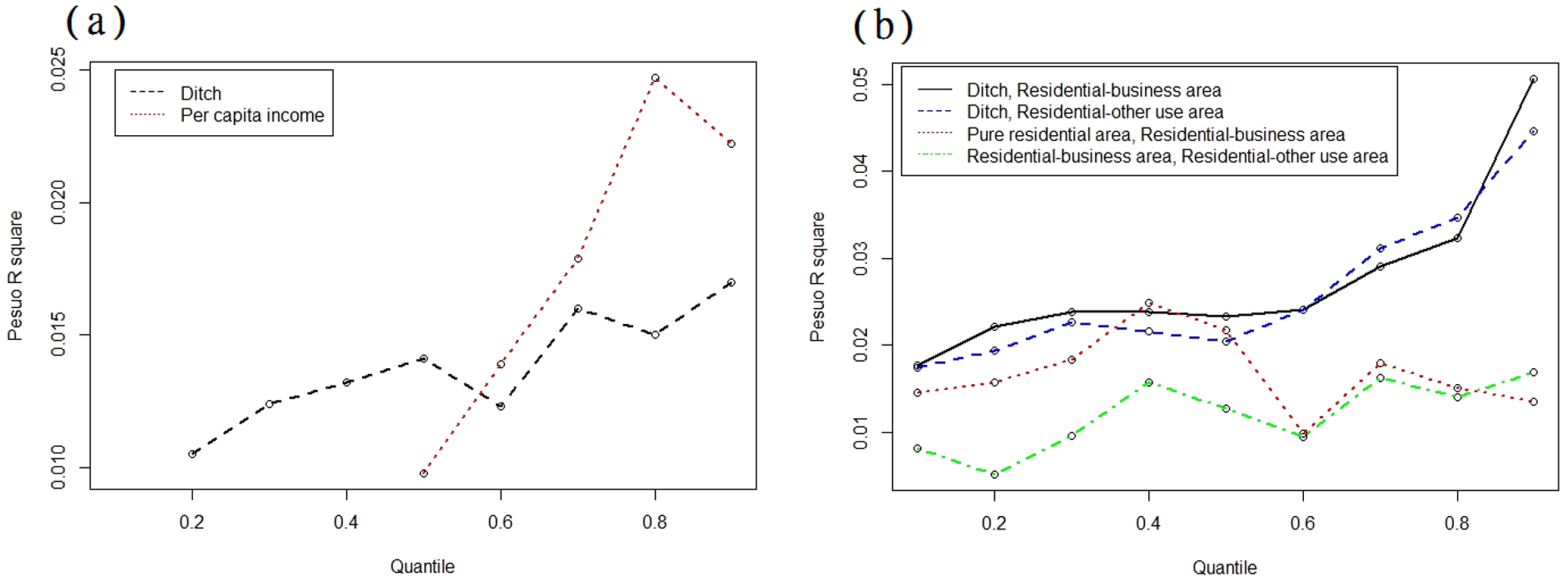
Variations of Pseudo R-square across quantile levels of (a) the two most significant risk factors, and (b) the four most significant interaction effects.

Associations between the spatial distributions of DF incidence rates and risk factors (i.e., land use and socioeconomic attributes) were estimated using quantile regression for every selected quantile level, 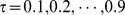. Before analysis, Pearson's rho was used to evaluate collinearity instead of the PCA because the *pR^2^* was below 10%. Following the covariate selection procedure, major DF risk factors for all quantiles were identified, including area ratios of canals, ditches, purely residential areas, residential areas with businesses, residential areas with other uses, warehouses, government agencies, elementary schools, high schools, and the socioeconomic factor, per capita income. Among these risk factors, the most significant were ditches and per capita income, indicating that the effect on DF risk can change among various quantiles (Fig. 4a). Spatial distributions of ditches and per capita income explained approximately 1% to 1.5% and 1% to 2% of DF variations, respectively.

**Figure 5:**
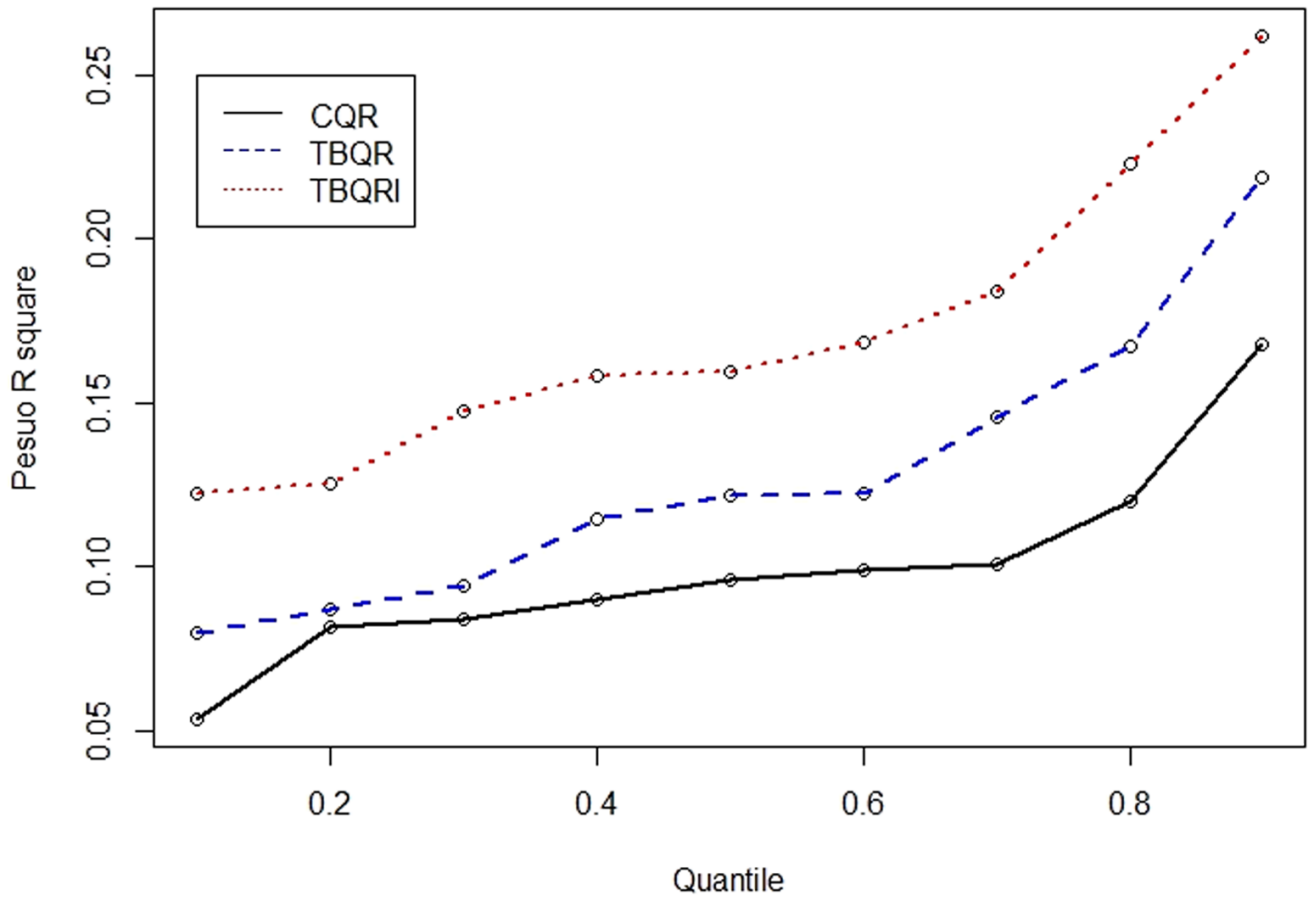
Comparison of pseudo R-squares across quantile levels among 1) conventional quantile regression model (CQR), 2) threshold-based quantile regression model (TBQR), and 3) threshold-based quantile regression considering interaction effects (TBQRI).

Furthermore, we considered interactions between the risk factors. Figure 4b shows the four most significant interaction factors, among which the interactions between ditches and residential areas contributed to changes in DF risk. Specifically, interactions between ditches and residential-business mixed areas and ditches and residential-other purpose mixed areas accounted for approximately 2% to 5% and 2% to 4%, respectively, of the DF spatial variability among quantile levels. Figure 5 depicts the *pR^2^* values in the selected quantile levels for the optimal models with three distinct settings (i.e., conventional quantile regression model, threshold-based quantile regression model, and threshold-based quantile regression that considers interaction effects). Consideration of the covariate threshold and interactions among risk factors can significantly improve the explanatory power of the model. In addition, all three models performed more effectively at higher quantile levels (i.e., areas of higher DF risk).

**Figure 6:**
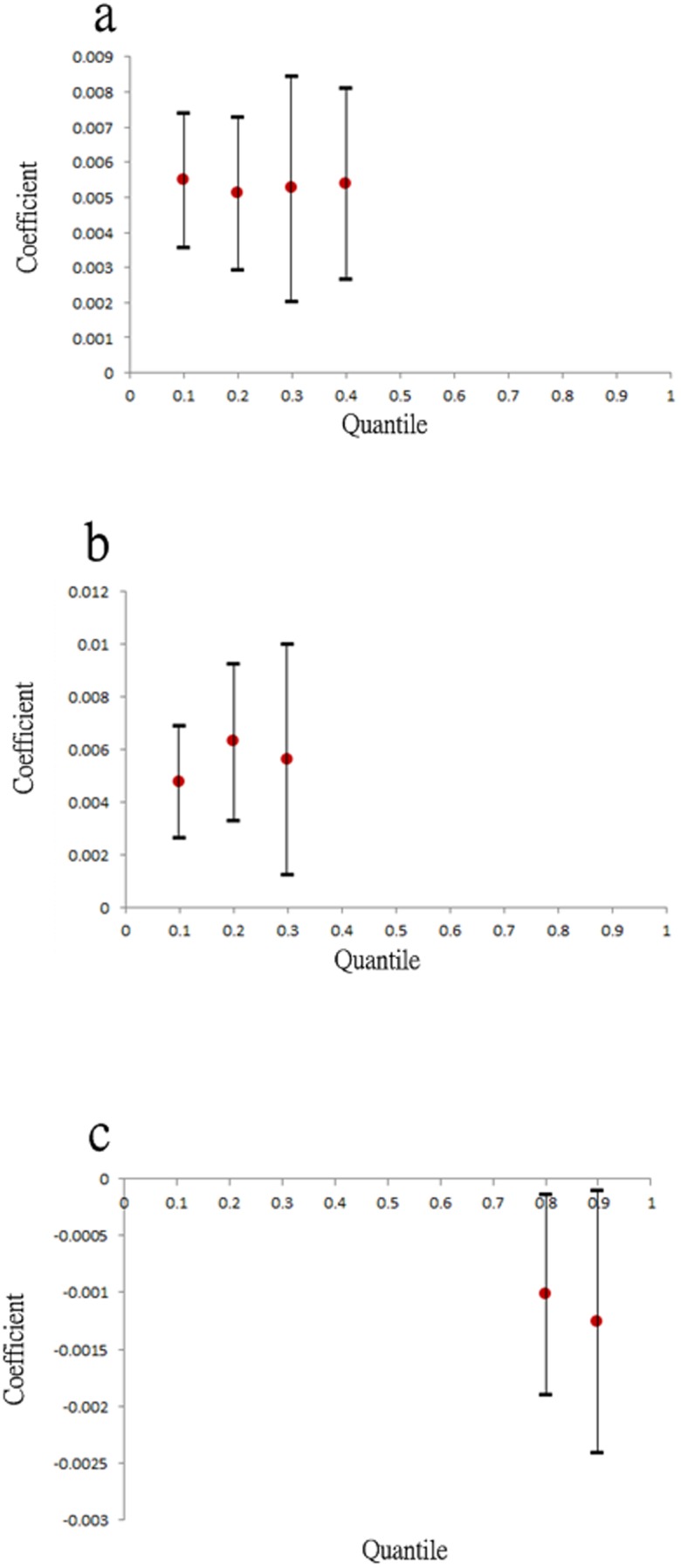
The 95% confident interval at the significant quantile levels for (a) canal (b) ditch, and (c) per capita income, respectively.

**Figure 7:**
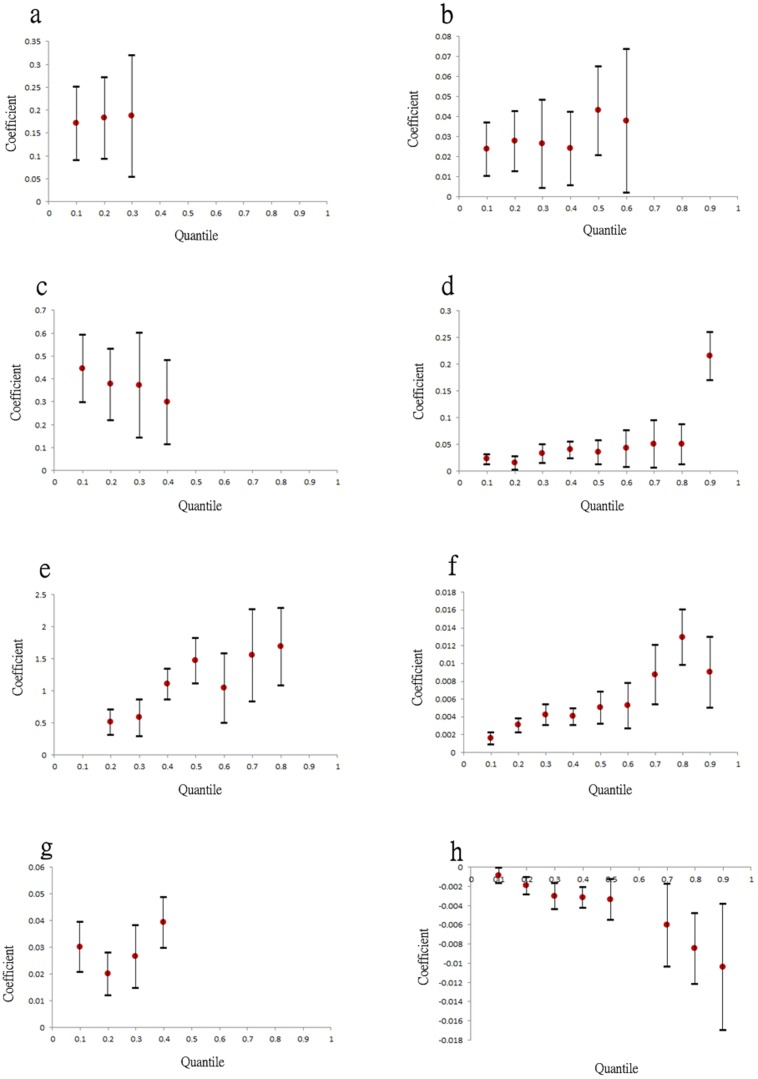
The 95% confident intervals at the significant quantile levels for interaction effects between (a) canal and ditches, (b) canal and residential-business area, (c) canal and residential-other use area, (d) ditch and residential-business area, (e) ditch and residential-other use area, (f) pure residential area and residential-business area, (g) residential-business area and residential-other use area, and (h) residential-business area and per capita income, respectively.

Figure 6 depicts the estimated coefficients and their corresponding 95% confidence intervals for the factors of canals, ditches, and per capita income, which significantly affected DF occurrence at various quantile levels. Among them, canals and ditches were critical to DF incidence in areas with lower incident rates (i.e., 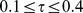). These land-use effects at the identified quantile levels were approximately constant, that is, a 1% increase in land-use ratios of canals or ditches elevated DF incidence rates by approximately 0.005%. By contrast, per capita income was negatively associated with DF, particularly at relatively high incidence rate quantile levels (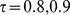). Figure 7 illustrates the identified critical interactions for DF incidence. Generally, the interactions between water-related utilities and residential areas were vital to the DF incidence rate spatial distribution and nonlinearly affected DF occurrence across the entire range of quantile levels, implying that the relationships between these interaction factors and DF risk can change significantly with the DF incidence rate level. Among the interactions, canal-related interactions with ditches, residential–business mixed areas, and residential–other-purposes mixed areas exerted significant effects at low-median quantile levels, (i.e., 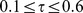). The ditch-related interactions with residential–business mixed areas and residential–other-purposes mixed areas exerted increasing effects when the DF incidence rate quantile levels increased. When residential–business areas were combined with purely residential areas, higher DF risk in relatively high quantile levels occurred compared with when they were combined with residential–other-purpose areas. Conversely, areas with high residential area per capita income reduced the effects of residential–business areas on DF risk, particularly at the high quantile levels.

**Figure 8:**
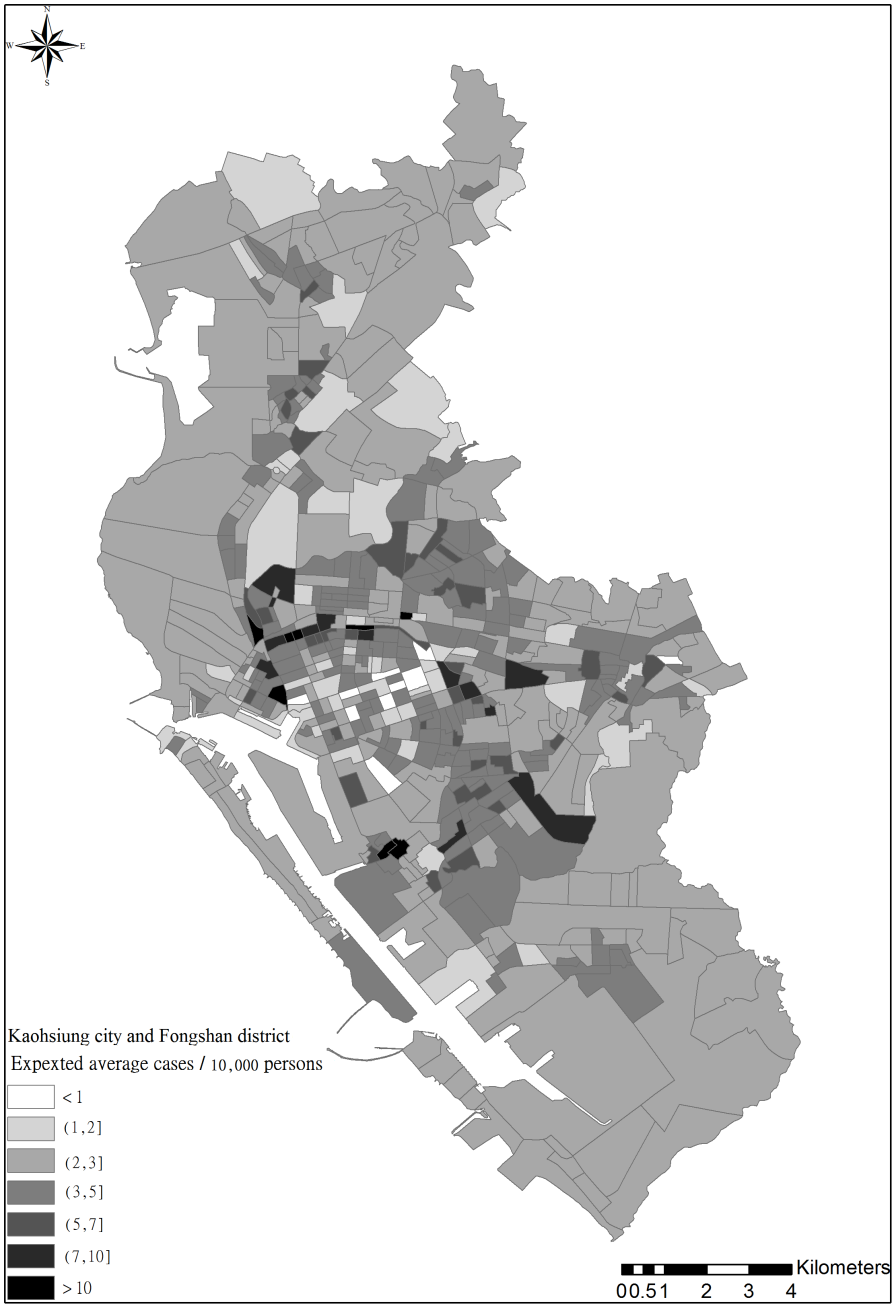
Spatial distribution of expected average incidence rates in the study area.

**Figure 9:**
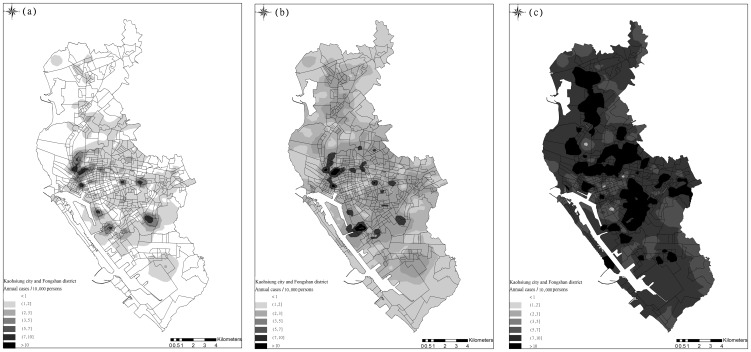
Spatial distribution of DF risks in terms of estimated incidence rates at return period of (a) 1.111 years, (b) 2 years, and (c) 10 years.

**Figure 10:**
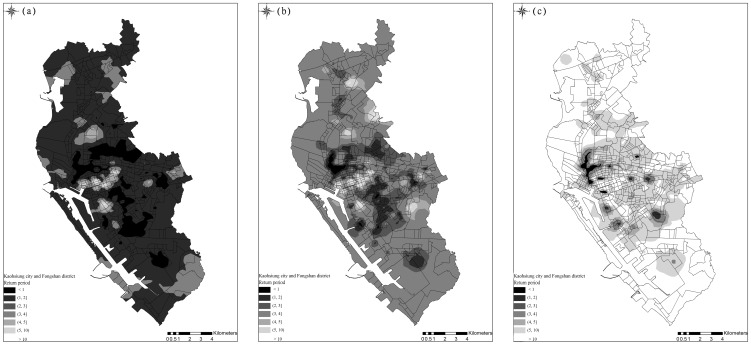
Spatial distribution of DF risks in terms of the estimated return periods that incidence rate is greater than (a) 2 cases/10,000 persons, (b) 1 case/10,000 persons, and (c) 8 case/10,000 persons.

The semiparametric CDF of DF incidence rates in the studied 535 *li* were estimated using the threshold-based quantile regression results. Figure 8 depicts the expected DF incidence rates across the area, based on the CDFs, similar to the observed average incident rates in Figure 2. In addition, Figures 9a–c show the incidence rate spatial distributions when the three selected exceedance probabilities were 0.9, 0.5, and 0.1, regarding the return periods (i.e., 1.11, 2, and 10 years). When the return period was 1.11 years, higher DF incidence rates occurred in areas close to canals or ditches. When return periods increased, the high risk areas shifted to areas with a mix of residential areas, canals, and ditches. Spatial distributions of the return periods at the three selected DF incidence cases are shown in Figures 10a–c. Approximately80% of the *li* had return periods shorter than 2 years when the annual DF incidence rate was one. As the selected annual DF cases increased, the *li* return periods also increased. The *li* with shorter return periods (i.e., 2–5 years) were located in areas with a mix of residential areas, canals, and ditches.

## Discussion

This paper proposed a novel probabilistic risk assessment approach to determine the DF risk spatial distributions, using quantile regression. Although quantile regression has been widely applied to analyze nonlinear functional relationships between quantities of concerns and covariates, according to an extensive review of the literature, this study is the first to integrate quantile regression and frequency analysis in probabilistic disease risk assessment. We further accounted for the potential threshold effects of DF occurrence risk factors and expanded conventional quantile regression into a threshold-based model. Using the threshold approach allows for the assumption that risk factors only take effect when they are larger than preselected values [43], [44] and is useful in environmental management and policy making [45], [46]. The proposed threshold-based quantile regression approach allowed for nonlinearities in both the relationships and predictor values. We not only provided probabilistic-based DF risk maps, but also identified the major risk factors affecting the spatial distribution of DF incidence rates, which were expressed as various return periods that presented the occurrence frequency of prescribed magnitudes. This presentation of DF risk can be interpreted and used to inform the general public and decision makers [29].

We considered land use and socioeconomic factors as proxies for DF disease modeling, because both of these factors are closely associated with DF vector habitats [47]–[49]. Land use patterns can indicate whether the environmental conditions of surrounding areas are favorable to vector breeding [50], and socioeconomic factors can signal human behavior and activities in the areas; both of these factors have been widely used for DF modeling [19], [20], [51], [52]. Unlike conventional approaches, linear models have been used extensively to identify significant risk factors [15], [16], [21], [53]. However, the associations between DF incidence and risk factors are not necessarily linear; the magnitudes of these associations depend on the levels of risk factors. We determined the varying associations between significant risk factors and DF incidence in areas with various epidemic conditions, using quantile regression. This study is the first to use quantile regression to analyze the functional relations between DF risk factors and incidence. A two-stage variable selection process (i.e., variable screening and stepwise variable selection) was used to reduce collinearity issues and identify the most influential and explainable factors in each DF quantile. Although other variable selection or reduction methods are available (e.g., principle component analysis), the proposed variable selection process was used to provide direct relationships between landuse and socioeconomic patterns and DF risk. Therefore, these findings can serve as a reference for governmental agencies when implementing disease control practices in the study area.

Areas including both residential areas and open water channels (e.g., canals and ditches) exhibited elevated DF risk, which is consistent with previous findings [14], [16], [22], [24], [39]. The findings in this study indicated that *Aedes aegypti* is the primary vector responsible for DF epidemics in Taiwan, even though both *Aedes aegypti* and *Aedes albopictus* coexist in the country [54], [55]. Studies have revealed that water-related facilities are closely associated with an abundance of *Aedes aegypti* [56], [57], particularly in areas with numerous water-holding containers [55], [58]. In addition, urban areas are generally the preferred habitat of *Aedes aegypti* [59], [60], an observation that supports the finding in this study that urbanization level was a highly influential factor that elevated DF risk [24]. The results further determined that residential areas with small businesses had higher DF risk than other types of residential area (e.g., purely residential areas; Fig. 7), possibly because of the relatively high water storage requirements for business activities (e.g., open-air food and grocery markets) [35], [36]. DF risk was further elevated by the existence of ditches in the area (Fig. 7). By contrast, high per capita income mitigated DF risk, particularly in residential–business areas in which banks or business offices were located on the lower floors of buildings. The approach used in this study emphasized the nonlinear contributions of environmental factors to DF occurrence that can be overlooked when using conventional regression methods. For example, the significance of water-related facilities to DF occurrence in the study area was only observed in lower quantiles (Figs. 6 and 7), a phenomenon that is easily overlooked when using conventional regression.

Distinct spatial patterns of DF incidence rates regarding various return periods indicated that areas close to ditches and downstream of major canals, (i.e., the Ai river) are generally DF epidemic foci. Figure 9 illustrates the areas with high DF incidence rates in the three selected return periods (i.e., 1.11, 2, and 10 years). These areas were exposed to high DF risk nearly every year, whereas downtown areas, such as Cianjhen District, have longer return periods (i.e., a high DF incidence in this area was observed only once every several years). These results suggested that open water channels in the city increase DF transmission, particularly in the areas downstream of the Ai river where both high-density residential and business areas are mixed. Clustered infections can easily occur in the high-density downtown residential areas once a DF epidemic has occurred. These finding provide insight into previous results that determined that urbanization in Kaohsiung City can increase the DF risk [24]. Another presentation shows the spatial distributions of return periods according to selected DF incidence rates. Most parts of the study area exhibited at least mild DF transmission (i.e., morbidity rate of 1/10000) every 3 years, except for Sinsing District. Areas with short return periods (e.g., 1–2 years), were restricted to areas downstream of the Ai river and those in Cianjhen District once the DF risk level increased, (e.g., 2/10000). The return period map can provide an alternative view for governmental agencies when estimating the frequency of and contributions to DF occurrence in the area. Canals and ditches are critical factors in the lower quantiles of DF occurrence in Kaohsiung City (Figs. 6 and 7), and therefore, are major risk factors for annual DF outbreaks. By contrast, residential areas were identified as the major factors in extreme DF outbreaks.

Risk analysis based on full probability densities of DF across the entire city can provide a multifaceted view of the associations between spatial distributions of environmental conditions and DF risk. This analysis can be used to emphasize the importance of considering nonlinear functional associations between environmental conditions and DF incidence at various quantile levels. Nonlinearity is crucial to the interpretation of the DF risk spatial distribution. However, the environmental conditions considered in this study (i.e., land use and socioeconomic factors) only partially explained the spatial characteristics of DF occurrence. More specifically, the factors considered in this study were only proxies to characterize the vector habitats and the interactions between humans and vectors. DF incidence can also depend on other direct or indirect risk factors, such as climatic variables, virus serotypes, imported cases, clustered transmissions, and disease control interventions [58], [61]. Consequently, the *pR^2^* of quantile regression models only accounted for approximately 25% of the space-time variations in the disease data. The spatial variation of climatic variables within the study area should have been limited, because of the small size and topographical changes. Other limitations resulting from data uncertainty should also be considered (i.e., the space-time observation scales varied between DF data and proxy factors). Land-use data for Taiwan were only available during 2007, and therefore, no land-use change was considered in this analysis during the study period. In addition, only country-scale socioeconomic data were available; therefore, this study did not include smaller-scale data (i.e., *li*). According to these data uncertainties, the probability and return period maps presented in this study can only provide spatial distribution patterns of DF risk rather than estimations of DF epidemic magnitude.
